# Evaluation of the stability and intratumoral delivery of foreign transgenes encoded by an oncolytic Foamy Virus vector

**DOI:** 10.1038/s41417-022-00431-y

**Published:** 2022-02-10

**Authors:** Karol M. Budzik, Rebecca A. Nace, Yasuhiro Ikeda, Stephen J. Russell

**Affiliations:** Department of Molecular Medicine, Mayo Clinic, Rochester, MN USA

**Keywords:** Genetic vectors, Cancer therapy

## Abstract

Foamy Viruses are cell cycle-dependent retroviruses capable of persisting unintegrated in quiescent cells until cell division occurs. This unique ability allows them to target slowly dividing human tumor cells which remains an unmet need in oncolytic virotherapy. We have previously reported the generation of oncolytic Foamy Virus (oFV) vector system and demonstrated its superiority over oncolytic Murine Leukemia Virus vectors in infecting slowly dividing cancer cells. In the present study we evaluated (i) the ability of oFV to carry foreign transgenes and (ii) the genetic stability of these vectors upon serial passage. The thymidine kinase (TK) and inducible caspase 9 (iCasp9) cDNAs could be detected in the oFV backbone for up to 3 in vitro passages. In vivo, GFP-, TK- and iCasp9- carrying oFV vectors propagated efficiently in subcutaneous xenograft glioblastoma tumors and drove transgene expression for up to 66 days. However, in vivo oFV vector spread eventually resulted in complete loss of the iCasp9 cDNA, minor loss of the TK cDNA and negligible loss of the GFP. Our results suggest that oFV is a promising gene delivery platform and that transgenes smaller than 1 kb might be most suitable for oFV arming.

## Introduction

Foamy Viruses (FV) are complex retroviruses grouped within the *Spumaretrovirinae* subfamily. Estimated to have existed for at least 450 million years, these ancient viruses have a long history of co-evolution with their natural hosts, which in the case of mammals has likely been ongoing for at least 100 million years [1, 2]. As a result, various FV serotypes circulate among different mammalian species without inducing any apparent pathology in their hosts [3–5]. The lack of pathogenicity in vivo stands in stark contrast to their in vitro infections which lead to strong cytopathic effects evidenced by vacuolization and extensive syncytia formation, hence their name—foamy viruses [6]. FVs are highly prevalent among their natural hosts as nonpathogenic agents, with rates of chimpanzee Simian FV (SFVcpz) infection ranging between 40 and 100% among adult chimpanzees [7]. SFVs from old world nonhuman primates (NHP) can be transmitted to humans occupationally exposed to NHP, such as bushmeat hunters and butchers, zookeepers, and laboratory personnel. Infected individuals, however, never develop any signs of disease, similar to natural hosts, and no active viral replication or human to human transmission has been reported so far [3, 8–13]. FVs only infect dividing cells, as integration of the FV genome is dependent on nuclear membrane disintegration [14]. However, in contrast to other retroviruses, FVs are uniquely capable of delaying uncoating in quiescent cells for weeks, providing physical protection of the FV genome from degradation until the cells start dividing and capsid degradation commences [15]. This feature potentially makes FVs more efficient at infecting and spreading in slowly dividing cells, such as human tumor cells, than their gammaretroviral cousins.

Toca 511 is a replication-competent Moloney Murine Leukemia Virus (MLV) vector armed with Cytosine Deaminase (CD) [16]. Combination therapy using Toca 511 with 5-Fluorocytosine (5-FC, an inactive prodrug that is converted by CD into the chemotherapeutic 5-Fluorouracil, 5-FU) showed very promising efficacy in murine models of cancer, however failed to show benefit in a phase three clinical trial for glioblastoma and anaplastic astrocytoma [17–21]. Molecular analyses of samples collected from Toca 511-treated patients enrolled in this study demonstrated low levels of proviral DNA in tumor samples, indicating low efficiency of tumor infection and spread of Toca 511 [22]. Given that human tumors on average grow much more slowly than mouse tumors used pre-clinically, with reported median tumor doubling times varying from 34.9 days to at least 1.4 years [23, 24], vectors dependent on active cell division must be able to wait for nuclear membrane disintegration to ensure productive human tumor cell infection. The MLV capsid, however, is relatively unstable and has a very short intracytoplasmic half-life of 5.5–7.5 h, which makes it unlikely that the extranuclear capsid-genome complex of Toca 511 will remain intact until nuclear membrane disintegration occurs in slowly dividing cells [25]. Toca 511, therefore, lacks the properties required to spread efficiently in human tumors, potentially explaining the low levels of proviral DNA in tumor samples recovered from patients treated with Toca 511.

Oncolytic viruses (OV) can be potent cancer cell killers but even with efficient intratumoral propagation they rarely infect all cells within a tumor [26]. Therapeutic transgenes with bystander killing effects can be incorporated into the OV backbones to facilitate killing of uninfected cancer cells. There are various arming strategies for OVs, one of which is the use of suicide genes encoding enzymes that convert nonactive prodrugs into cytotoxic metabolites [27]. Thymidine Kinase (TK) of the Herpes Simplex Virus Type 1 phosphorylates Ganciclovir (GCV) into GCV monophosphate, which is then further phosphorylated by cellular kinases into GCV-triphosphate (GCV-TP). GCV-TP is a toxic analogue of guanosine triphosphate. When incorporated into the replicating DNA chain, GCV-TP leads to DNA chain termination, single-strand breaks and eventually cell death [28]. Unlike TK, whose mechanism of action relies on disrupting DNA synthesis and, therefore, cell division, inducible caspase 9 (iCasp9) can directly activate apoptotic pathways of the cell, regardless of its mitotic capabilities. Upon mitochondrial membrane disruption, cytochrome C leaks into the cytoplasm, which leads to the activation of caspase 9 and its dimerization. Dimerized caspase 9 then activates downstream cascade of caspases, which ultimately leads to apoptosis [29]. iCasp9 is a caspase 9 fused with the human FK506 binding protein (FKBP), which dimerizes upon addition of a synthetic dimerizer such as AP1903 or AP20187, therefore artificially inducing dimerization of caspase 9 and apoptosis [30].

Genetic stability of replicating viral vectors is crucial for efficient therapeutic transgene distribution within diseased tissues during viral spread. Replication-competent retroviral vectors have often proven to be unstable, with some vectors losing their transgenes within one in vitro virus passage [31]. Transgene insertion might interfere with retroviral replication by disrupting regulatory sequences or impacting the secondary or tertiary structural elements of the viral RNA governing various aspects of the viral life cycle. Mutant vectors with their genetic cargo lost due to deletion events may have a selective advantage that allows them to replicate more efficiently than viruses with intact transgene sequences. As a result the transgene-deleted variants outgrow the parental vectors [32], limiting transgene distribution within the infected tissue.

We previously reported the generation of a chimeric replication-competent oncolytic FV (oFV) vector with components derived from two strains of SFVcpz and demonstrated its potent oncolytic activity in vivo as well as a more efficient spread in slowly dividing human tumor cells than an MLV vector [33]. The purpose of the current study was to evaluate the ability of oFV to deliver various transgenes into tumors and facilitate their expression, as well as investigate the stability of the transgene-carrying vectors. Green fluorescent protein (GFP), TK and iCasp9, were expressed upon both in vitro and in vivo infection. GCV or AP20187 treatment led to robust killing of various human and mouse cancer cell lines infected with oFV-TK or oFV-iCasp9, respectively, both in vitro and in vivo, in subcutaneous glioblastoma tumors. oFV-GFP, -TK and -iCasp9 extensively propagated in the tumors, as demonstrated by serial luminescence imaging of the infected mice (the tumor cell line had been programmed to express firefly luciferase (Fluc) and red fluorescence protein mCherry in response to oFV infection). Analysis of the stability of the oFV vectors upon long-term in vivo amplification revealed that transgene loss by oFV-GFP was negligible, partial for oFV-TK, and complete for oFV-iCasp9. Our results indicated that oFV vectors are capable of delivering various transgenes into tumors in vivo facilitating their long-term expression, however the stability of the carried sequences varies and appears to be sequence dependent.

## Results

### Generation of transgene-carrying oFV vectors and indicator glioblastoma cells for non-invasive monitoring of oFV replication in vivo

**Fig. 1:**
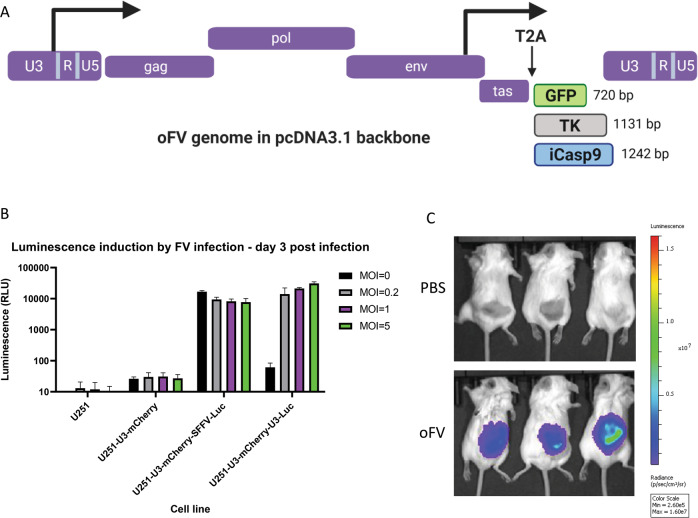
Structure of the transgene-carrying oFV vectors and functional analysis of the indicator U251 cells expressing firefly luciferase in response to FV indection. **A** Structure of the transgene-carrying oFV vectors used in the study.TK – HSV-1 thymidine kinase, iCasp9—inducible caspase 9, T2A—T2A self-cleaving peptide. **B** indicator U251-U3-mCherry-U3-Luc cells express firefly luciferase only in response to FV infection. Wild type U251, U251-U3-mCherry (expressing mCherry under the FV U3 promoter), U251-U3-mCherry-SSFV-Luc (expressing mCherry under the FV U3 promoter and firefly luciferase under the constitutive SFFV promoter), U251-U3-mCherry-U3-Luc (expressing both mCherry and firefly luciferase under the FV U3 promoter) were infected with oFV at indicated MOIs. 3 days postinfection the luciferase assay was performed to determine the luciferase expression in the infected cells. The experiment was performed in duplicate in two independent experiments. U3—FV promoter-containing region from the viral LTR, Luc- firefly luciferase. **C**. Subcutaneous indicator U251-U3-mCherry-U3-Luc tumors express firefly luciferase in response to oFV infection and the bioluminescence was imaged using IVIS Xenogen. Tumors were directly injected with 2 doses of 1 * 10^6^ IU of the parental oFV or PBS control and imaged 29 days postinfection.

To evaluate the ability of oFV to carry different therapeutic and non-therapeutic transgenes, we inserted cDNAs of various sizes into the oFV backbone. The three transgenes used in this study varied in size from 720 bp (GFP) to 1242 bp (iCasp9) (Fig. 1A). The generation of the oFV-GFP vector has been described previously [33]. The TK and iCasp9 carrying oFV vectors were generated by the replacement of the T2A-GFP cassette of oFV-GFP (flanked with SacII and BspEI restriction sites) with TK or iCasp9 cDNAs. The T2A self-cleaving peptide sequence was inserted upstream of the cDNAs to facilitate processing of the Tas-cargo fusion protein (Fig. 1A). Previously, we also described the generation of a FV indicator cell system allowing for Tas-dependent expression of mCherry in cells infected with oFV. Tas is a transactivator protein of FVs expressed from the ubiquitously active internal promoter. It activates the expression of the viral 5’ Long Terminal Repeat (LTR) promoter located in the U3 element of the LTR [34]. To facilitate non-invasive monitoring of oFV replication in tumors in vivo, we generated human glioblastoma U251 cells carrying both mCherry and Fluc reporter genes under the U3 region of PAN1 SFVcpz. Infection of U251 cells with oFV led to a dose-dependent expression of firefly luciferase only when the cells had previously been transduced with a lentviral vector carrying U3-driven Fluc (Fig. 1B). The U251-U3-mCherry-U3-luciferase cell line allowed for easy and reliable detection of oFV infected cells in established subcutaneous tumors via fluorescent imaging of mCherry as well as non-invasively using the Xenogen IVIS system (Fig. 1C).

### GCV and AP20187 treatment accelerates death of cancer cells infected with oFV-TK and oFV-iCasp9

**Fig. 2:**
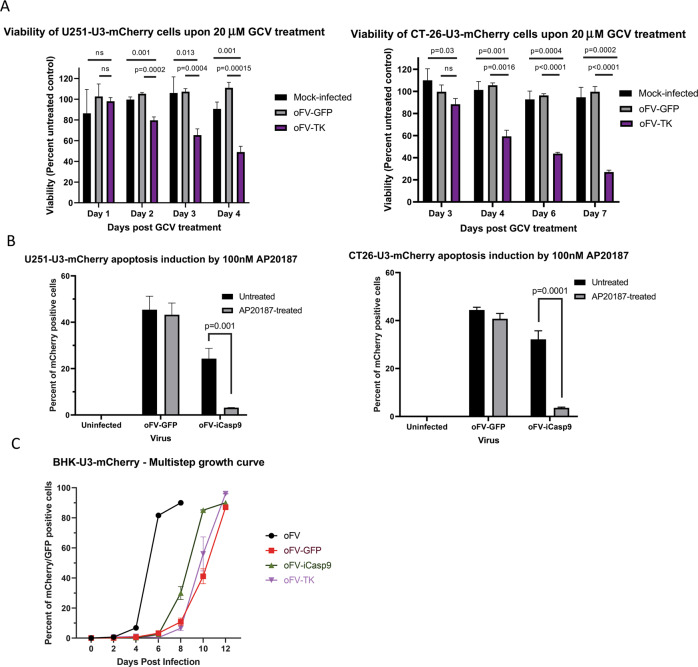
Encoding suicide genes in the oFV backbone accelerates cancer cell death in vitro upon prodrug treatment. **A** Viability of indicator U251-U3-mCherry and CT-26-U3-mCherry cancer cell lines mock infected or infected with oFV-GFP or oFV-TK at MOI = 1 and treated with 20 μM GCV or mock control (results presented as percent of mock-treated control, experiment performed in triplicates, statistical significance determined using the Holm-Sidak method), **B** Percent of mCherry positive cells after mock, oFV-GFP or oFV-iCasp9 infection at MOI = 1 and treatment with AP20187 or mock control in indicator U251-U3-mCherry and CT-26-U3-mCherry cancer cell lines (measured 24 hours post AP20187 treatment, statistical significance determined using the Holm-Sidak method, performed in triplicates). **C** Spread of oFV, oFV-GFP, oFV-TK and oFV-iCasp9 in BHK-U3-mCherry cells (MOI = 0.01).

The transgene-carrying vectors were rescued as replication-competent and transgene expression in infected cells was analyzed. Mouse and human cancer cell lines, including CT-26 (mouse colorectal carcinoma), U251 (human glioblastoma) (Fig. 2A), SKOV-3 (human ovarian carcinoma) and HT-29 (human colorectal carcinoma) (Supplementary Fig. 1b) were infected with oFV-TK or control oFV at MOI = 1, or mock control. On day 3 (U251), 4 (CT-26), or 5 (SKOV-3 and HT-29) postinfection the cells were treated with GCV at 20 μM or mock control (DMSO). The treatment led to a visible decrease in the number of mCherry positive oFV-TK-infected indicator U251-U3-mCherry cells, which was not observed for the control oFV vector (Supplementary Fig. 1a). Viability of the infected cancer cells at different time points post-GCV or mock treatment was analyzed and calculated as percent of viability of the mock control (Fig. 2A, Supplementary Fig. 1b). In all the tested cell lines GCV treatment led to a decrease in viability exclusively in the oFV-TK infected group and the viability drop to ~30% of an untreated control, at which virtually all cells in the well were dead (the fluorescence signal of the viability dye in those wells was close to the background signal), occurred between day 4 and 7 post-treatment, depending on the cell line (Fig. 2A, Supplementary Fig. 1b).

Expression and function of the iCasp9 transgene in oFV-iCasp9-infected cells was analyzed in an analogous way. Indicator CT-26, U251 and HT-29 cells were infected with oFV-iCasp9, oFV-GFP or mock control at MOI = 1 and 3–5 days postinfection the cells were treated with the dimerizer AP20187 at 100 nM or left untreated. 24 h post-treatment we observed clear cytotoxicity in the oFV-iCasp9-infected U251-U3-mCherry cells (Supplementary Fig. 1c), and quantified the percentage of the infected, mCherry positive cells in all the tested cultures (Fig. 2B, Supplementary Fig. 1d). AP20187 treatment led to an almost complete elimination of the mCherry positive cells exclusively in the oFV-iCasp9 infected group. These results proved that both TK and iCasp9 are functionally expressed from the oFV backbone. Subsequently, we determined the growth dynamics of the armed oFV vectors in comparison to oFV-GFP and the parental oFV virus (Fig. 2C). We infected BHK-U3-mCherry cells with oFV, oFV-GFP, -TK and -iCasp9 at MOI = 0.01 and assessed the spread of the viruses in the cultures by flow cytometry to determine the percentage of infected, mCherry positive cells every 2 days. The analysis revealed that the armed viruses replicated similarly to oFV-GFP and the larger size of the therapeutic transgenes did not further attenuate the vectors.

### Armed oFV vectors propagate extensively in subcutaneous indicator glioblastoma tumors, GCV and AP20187 treatment leads to the death of oFV-infected tumor cells

**Fig. 3:**
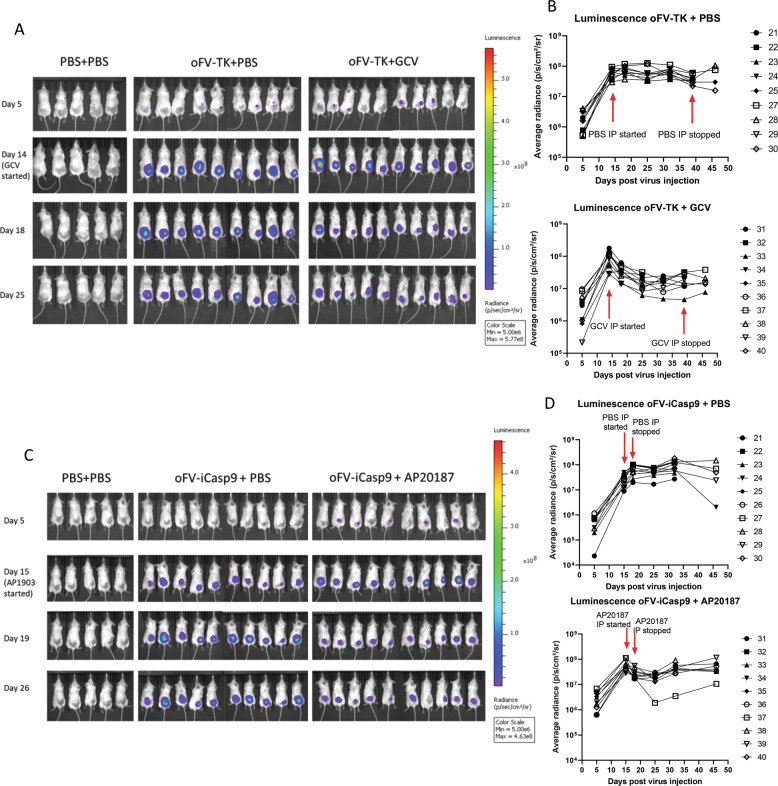
oFV vectors carrying therapeutic transgenes efficiently propagate in subcutaneous indicator U251-U3-mCherry-U3-Luc tumors and facilitate expression of the transgenes. Subcutaneous U251-U3-mCherry-U3-luc tumors were injected with five doses of 2 * 10^6^ IU of oFV-TK, oFV-iCasp9 or PBS control every 2 days, 14 days after the first infection the mice were injected intraperitoneally with GCV at 50 mg/kg daily for four five-day cycles, AP20187 at 5mg/kg daily for four days or mock control, **A.**, **C**. bioluminescence imaging of the tumor bearing mice infected with oFV-TK (A) and oFV-iCasp9 (**C**). **B., D**. Quantification of the average radiance for each mouse in the experiment infected with mice infected with oFV-TK (**B**) and oFV-iCasp9 (**D**). GCV/AP20187 treatment leads to a decrease in luminescence in the oFV-TK/oFV-iCasp9 infected indicator tumors. Red arrows indicate the time points at which GCV/AP20187 treatment was started/stopped.

Next, we verified the spread and function of our armed oFV vectors in tumors in vivo. To that end, we implanted the indicator U251-U3-mCherry-U3-luciferase cells subcutaneously in CB17 SCID mice and upon tumor establishment we infected them intratumorally with 5 doses of 2 * 10^6^ IU of oFV-TK, oFV-iCasp9 or PBS control. Both viruses spread efficiently in the infected indicator tumors as demonstrated by serial bioluminescence imaging of the infected mice (Figs. 3A, C). In the absence of GCV or AP20187, after peaking around day 20 post first virus injection, the bioluminescence plateaued and remained relatively unchanged until the last imaging (on day 46 post first virus injection) (Figs. 3B, D, top panels). When the infected mice were intraperitoneally treated with GCV at 50 mg/kg or AP20187 at 5 mg/kg, we observed a decrease in bioluminescence, which was especially profound for the oFV-TK/GCV combination (Figs. 3B, D, bottom panels). Interestingly, in the tumors treated with the oFV-iCasp9/AP20187 combination the decrease was only transient and the bioluminescence then increased, possibly due to ongoing multiplication of tumor cells carrying an integrated oFV provirus but not expressing a functional iCasp9 protein. Nevertheless, this experiment proved that both transgenes are expressed and functional upon oFV vector infection of tumors in vivo, although GCV and AP20187 treatment do not eliminate all the oFV-TK and oFV-iCasp9 infected tumor cells, respectively.

### Therapeutic transgenes in the oFV backbone are stable for up to 3 in vitro cell free passages

**Fig. 4:**
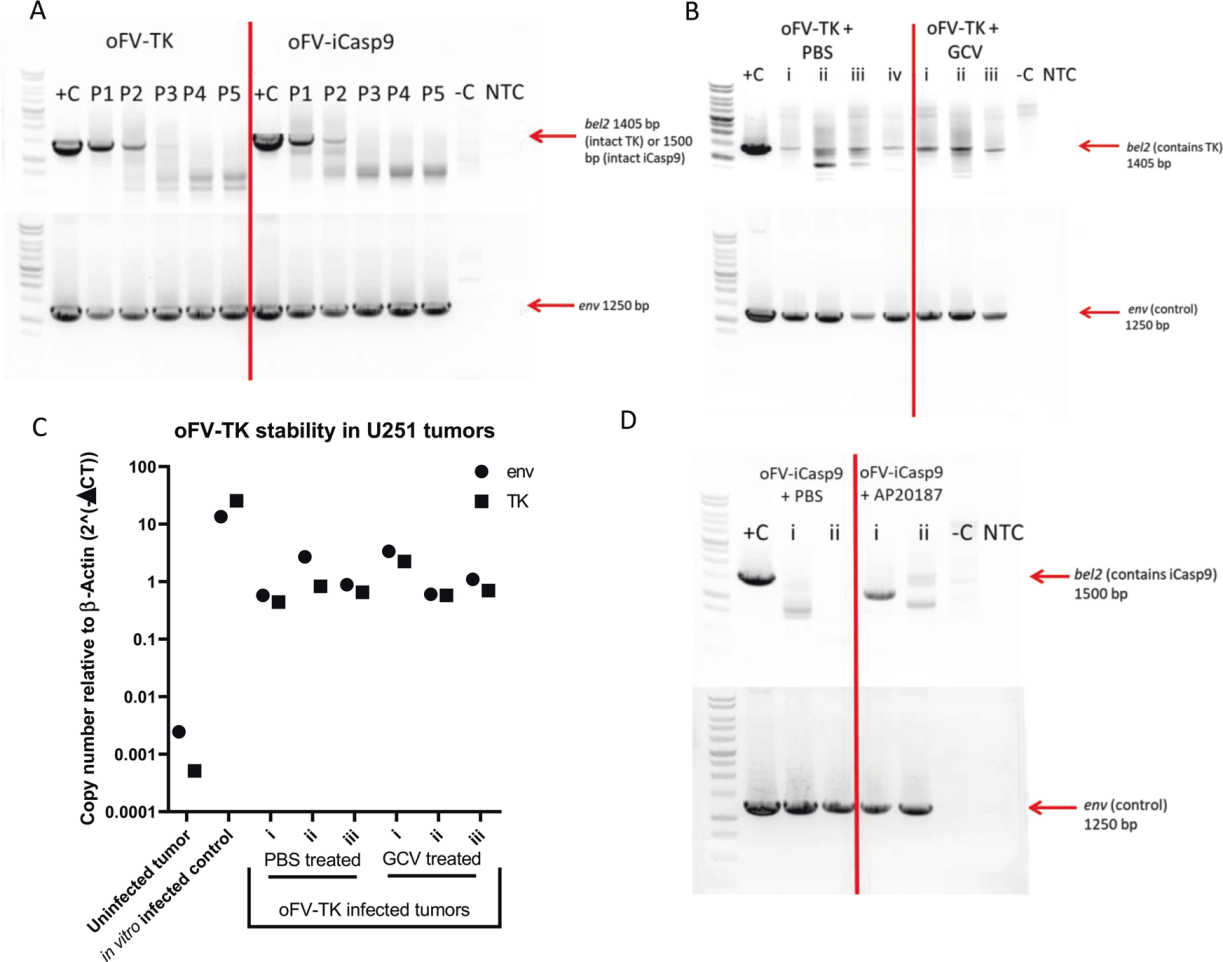
Therapeutic transgenes in the oFV backbone are stable for up to 3 in vitro passages, TK cDNA appears to be more stable than iCasp9 cDNA in the oFV backbone during in vivo amplification. **A** oFV-TK and oFV-iCasp9 were serially passaged five times at a fixed MOI = 0.5 in U251-U3-mCherry-U3-luc cells and at the end of each passage (day 8 postinfection) the genomic DNA was isolated from the infected cells for a PCR amplification of the *bel2* region (containing the transgene) or *env* (control). **B.**, **D**. U251-U3-mCherry-U3-luc tumors from mice infected with oFV-TK/oFV-iCasp9 and treated with GCV (*n* = 3)/AP20187 (*n* = 2) or PBS control (*n* = 3 for oFV-TK and *n* = 2 for oFV-iCasp9) were explanted and cultured in vitro, then genomic DNA was isolated from the explants and PCR analyzed for the presence of the oFV-TK (**B**) or oFV-iCasp9 (**D**) provirus (*env*) as well as the intact transgene cDNA (*bel2* region), **C**. qPCR reaction was performed on the DNA isolated from oFV-TK infected tumors to determine the *env* and TK copy number, result is normalized to endogenous β-actin copy number. +c – positive control (oFV-TK or –iCasp9 infectious clone DNA), -**C** – negative control (genomic DNA from uninfected U251-U3-mCherry-luc cells), NTC- no template control, P1-P5 – passage number, i-iv – specific tumors collected from the treated mice for analysis.

One possible explanation for the incomplete killing of tumor cells infected with the armed oFV vectors in vivo upon treatment with GCV and AP20187 could be drug insensitivity caused by transgene loss. We therefore analyzed the stability of the transgenes carried by oFV-TK and oFV-iCasp9 in vitro, upon serial passaging, and upon in vivo amplification. U251-U3-mCherry cells were infected with oFV-TK or oFV-iCasp9 at MOI = 0.5. On day 8 postinfection the media from the infected cells was filtered and the virus in the media was titrated. The titrated supernatant was used for a subsequent infection of U251-U3-mCherry-U3-luc cells at MOI = 0.5. This process was done for a total of 5 passages. At the end of each passage the infected cells were collected, total DNA isolated and analyzed by PCR for the presence of proviral DNA (using primers binding within the *env* gene) and the transgene (using primers that bind upstream and downstream of the transgene insertion site, within the retained sequences of *bel2*). While a prominent PCR product for a fragment of the oFV *env* gene (of 1250 bp size) was detected for both vectors in cells from every passage with complete absence of drop-out bands, the intact PCR product for the transgenes (1405 bp for TK and 1500 bp for iCasp9) was only detected for up to 3 passages (Fig. 4A). For oFV-TK, drop-out bands indicating deletion events within the TK cDNA became visible in passage two, and from passage 3 to 5 they were the dominant bands in the PCR product. Deletions in the iCasp9 cDNA occurred earlier than in the TK cDNA, as drop-out bands were visible already in the first passage and were the dominant product from passage 3 onward (Fig. 4A).

To further investigate the deletion events occurring in the transgenes carried by oFV vectors, we purified the deletion bands, cloned them using the TOPO cloning technology, and sequenced. The sequencing revealed that deletions occur exclusively in the transgene cDNA, never within viral sequences (Supplementary Fig. 2a, 2b). In addition, every clone displayed unique deletion sites, indicating that the deletions occur in a random manner and no discernable pattern is followed during the process.

### Therapeutic transgenes show variable stability upon in vivo amplification

To confirm the presence of the oFV provirus and determine the integrity of transgene cDNAs in the infected U251-U3-mcherry-U3-Luc tumors, we explanted tumors from the vector-infected and GCV/AP20187- or PBS-treated mice and expanded the cells in vitro. The tumors were harvested between 42 and 46 days postinfection (oFV-TK-infected tumors) or 49 days postinfection (oFV-iCasp9-infected tumors). The in vitro expanded cells were either (i) GCV/AP20187 or mock-treated and imaged via fluorescence microscopy, or (ii) collected for DNA isolation and subsequent PCR analysis. GCV treatment of the explanted tumors infected with oFV-TK led to killing of the infected, mCherry expressing indicator tumor cells, regardless of whether they originated from a PBS or a GCV-treated mouse (Supplementary Fig. 3a). PCR analysis of the in vitro passaged viruses revealed that oFV-TK provirus was present in all the tested tumors, as demonstrated by the prominent and intact *env* PCR product (Fig. 4B). A PCR product band corresponding to intact TK cDNA was found in all the analyzed oFV-TK infected tumors, however drop-out bands were present in all the tumor samples, albeit fainter than the 1405 bp band. Additionally, the banding pattern varied between different tumor samples (Fig. 4B). We then sequenced the drop-out bands, which revealed that the deletion events were random and occurred exclusively in the TK cDNA (Supplementary Fig. 3b), similar to our in vitro observations. To estimate the extent of transgene loss in the DNA from the explanted tumor samples we performed a qPCR reaction to quantify the TK cDNA and *env* copy numbers (Fig. 4C). The analysis revealed that TK to *env* copy numbers were very similar in all the tumors except for tumor ii from a oFV-TK/PBS treated mouse, which had a substantially lower TK than *env* copy number. Overall, these results indicated that while functional TK is present in the samples and the majority of proviral sequences in the tumors might still contain the transgene, the TK cDNA is partially lost upon amplification in vivo.

AP20187 treatment of explanted oFV-iCasp9-infected tumors did not result in any visible cell-killing effect (Supplementary Fig. 4a). PCR analysis of the DNA isolated from the explanted tumor cells revealed that the oFV-iCasp9 provirus was present in all the tested tumors, however, no intact iCasp9 cDNA was detected in these samples (Fig. 4D). Sequencing analysis of the drop-out bands revealed deletion events occurring in a random manner exclusively in the iCasp9 cDNA (Supplementary Fig. 4b). These finding suggested that iCasp9 cDNA is less stable than TK in the oFV backbone and is deleted during in vivo amplification.

### oFV-GFP extensively propagates in subcutaneous indicator glioblastoma tumors in vivo and facilitates long term intratumoral transgene expression without signs of significant transgene loss

**Fig. 5:**
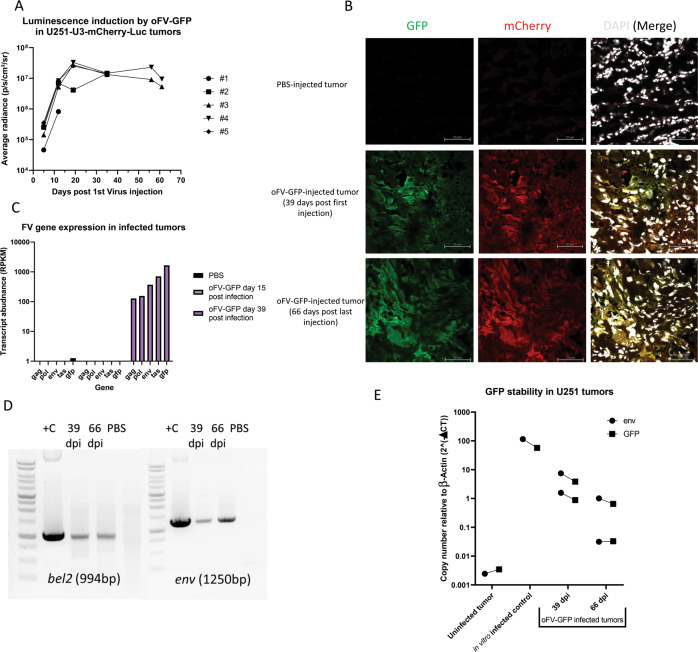
oFV-GFP extensively propagates in subcutaneous indicator glioblastoma tumors in vivo and facilitates long term intratumoral transgene expression without signs of significant transgene loss. 5 million U251-U3-mCherry-U3-luc cells were implanted subcutaneously in CB17-SCID mice and infected intratumorally with PBS control or 4 doses of 5 * 10^*5*^ IU oFV-GFP, **A** Average oFV-GFP-induced radiance in the oFV-GFP-infected tumors, **B** Immunofluorescence staining for mCherry and GFP in sections of PBS-treated and oFV-GFP infected tumors at different time points postinfection (mCherry expression is a marker of oFV-infected indicator tumor cells, GFP is a marker of oFV-GFP infection), **C**. Relative abundance of oFV-GFP transcripts in control and oFV-GFP-infected tumors determined with RNAseq. **D** Total DNA was isolated from oFV-GFP infected tumors harvested 39 (*n* = 2) and 66 (*n* = 2) days postinfection, as well as a PBS-treated tumor. The DNA was PCR analyzed for the presence of proviral (*env*) and GFP transgene (*bel2*) DNA. +C – positive, oFV-GFP infectious clone was used as template for the PCR reaction, 39 and 66 dpi – DNA from oFV-GFP infected tumors harvested 39 and 66 days postinfection, PBS- DNA from a PBS treated tumor. **E**. qPCR reaction was performed on the DNA isolated from the oFV-GFP infected and PBS treated tumors, as well as U251-U3-mCherry-U3-luc cells infected in vitro at MOI = 1 (control) to determine the *env* and GFP copy number, result is normalized to endogenous β-actin copy number.

To evaluate the intratumoral spread and stability of an oFV vector carrying a small, non-therapeutic transgene in tumors in vivo, we injected U251-U3-mCherry-U3-Luc tumors with 4 doses of 5 * 10^5^ IU oFV-GFP. The virus efficiently propagated in the infected tumors as demonstrated by increase of the bioluminescence signal in the infected indicator tumors over time (Fig. 5A, Supplementary Fig. 5A) as well as increase in viral gene expression over time (Fig. 5C). The infected tumors were strongly bioluminescent and mCherry-positive for at least 60 days postinfection (Figs. 5A, B), which together with our analysis of FV transcript abundance (Fig. 5C) indicated that oFV genes are actively expressed in these tumors. The expression of GFP was confirmed at the transcript level (Fig. 5C), as well as protein level (Fig. 5B) in tumors harvested at 15 to 66 days postinfection. mCherry, the marker of oFV-infected cells, strongly co-localized with GFP in the analyzed tumors, indicating that GFP might be stable in the viral backbone at least 66 days (Fig. 5B). Areas positive only for mCherry were found in those tumors (Supplementary Fig. 5b), however, they were very rare. To further investigate the stability of the GFP cDNA in the oFV-GFP vector, we isolated chromosomal DNA from oFV-GFP infected tumors harvested on day 39 or 66 postinfection. A PCR reaction was then performed on the chromosomal DNA to amplify the *bel2* region, which contains the GFP cDNA, and a fragment of *env* (Fig. 5D). In contrast to oFV-TK and iCasp9, PCR-amplification of *bel2* in the DNA from the oFV-GFP-infected tumors did not result in any visible drop out bands. A qPCR copy number quantification for GFP and *env* revealed that in all the infected tumors the *env* to GFP copy numbers were very similar, just like in in vitro infected cells (Fig. 5E). This analysis demonstrated that the GFP cDNA is more stable than TK or iCasp9 during in vivo amplification of oFV vectors.

## Discussion

Preclinical studies with replicating retroviral vectors (RRV) have shown encouraging results in the treatment of mouse models of cancer [17, 19, 35, 36], however, the slow growth of human tumors might create a significant barrier for the use of gammaretroviral vectors in the clinical setting, as their infection and spread is highly dependent on active cell proliferation. We recently reported the generation of a new RRV system based on SFVcpz (oFV) and demonstrated that despite its infection being dependent on cell proliferation, oFV can circumvent the issue of slow tumor growth [33]. oFVs spread more efficiently than MLV-based vectors in slowly dividing tumor cells as, in contrast to the MLV vectors, they are capable of latently persisting in quiescent cells until cell division can occur [33]. Though efficient intratumoral propagation of an OV is essential for the efficacy of OV therapy, it is unlikely to result in infection of 100% of cells within a tumor [26]. Expression of proteins that can directly or indirectly induce killing of unifected cells can potentially circumvent this issue and enhance the OV-mediated tumor destruction. We therefore verified the ability of oFV to carry transgenes of various sizes, ranging from 720 (GFP) to 1242 (iCasp9) bp, including two therapeutic transgenes: HSV-1 TK and iCasp9. Our analyses revealed that the expression of the transgenes carried by oFV-TK, oFV-iCasp9 and oFV-GFP was successfully confirmed after both in vitro and in vivo infection (Figs. 2A, B, 3, 5B). cDNAs of the TK and iCasp9 transgenes could be detected for up to 3 in vitro passages of the oFV vectors in U251-U3-mCherry-U3-luc cells (Fig. 4A). Interestingly, after 42 to 46 days of oFV-TK amplification in subcutaneous U251-U3-mCherry-U3-luc tumors in vivo majority of the proviruses detected in the chromosomal DNA isolated from the infected tumor cells still carried intact TK cDNA (Figs. 4B, C), indicating minor transgene loss. During a 49 day period of intratumoral oFV-iCasp9 vector amplification, iCasp9 cDNA was completely lost as we could not detect intact iCasp9 cDNA in the proviral genome sequences detected in the chromosomal DNA isolated from infected tumor cells (Fig. 4D). In contrast, oFV-GFP propagation in tumors drove intratumoral transgene expression for at least 66 days with no signs of substantial GFP cDNA loss (Fig. 5).

RRVs based on MLV carrying transgenes in their LTR have been reported to be unstable, losing their transgene expression over two in vitro passages and sometimes already during vector production [31, 32]. It was hypothesized that transgene size might impact stability, as smaller transgenes (of about 900 bp) were carried more stably than larger cargo [37]. Later, a replicating MLV vector carrying GFP cDNA located immediately downstream of *env* and translated from an IRES was described and reported to facilitate stable expression of the transgene for at least 7 in vitro passages [38]. This vector was ultimately developed as the cancer therapeutic Toca 511 [16]. The higher stability of the Toca 511 vector suggests that foreign sequences in certain positions in the viral genome might interfere with viral replication without directly inactivating the virus, which results in selective deletions within the burdensome genetic cargo.

Prototype Foamy Virus (PFV) based RRVs carrying foreign transgenes in the place of a fragment of the *bel2* gene, similarly to our oFV vectors, have been described previously [39, 40]. The stability of the cargo in these vectors was variable, with some transgenes stably expressed after 10 in vitro passages (purine nucleoside phosphorylase, 720 bp size), while other were lost after 5 passages (TK) [39]. The deletions observed for the less stable transgenes were unlikely to have been caused by a limited packaging capacity of the PFV capsid as replication-defective PFV vectors were recently shown to efficiently package genomes giving rise to proviral sequences of 15 kb [41], while the size of the TK encoding PFV proviruses did not exceed 13.5 kb. Moreover, the transgene size did not directly impact the stability of the replicating PFV vectors as a 1.3 kb transgene was more stable than 1.1 kb cargo [39, 40]. Therefore, given the replication-competent nature of these vectors, it is possible that the virus preferentially loses sequences not required for its replication and that some foreign sequences might interfere with the viral life cycle more than others, thus parental vectors are outcompeted more rapidly.

In vitro passaging of our oFV vectors led to a rapid selection of deletion variants that had lost transgene cDNAs from the viral genome, both in the case of suicide gene armed vectors (Fig. 4A) and in the previously described oFV-GFP vector [33], whereas the TK and GFP cDNAs appeared to be more stable during in vivo amplification (Figs. 4B, C, 5B, C, D). It is possible that the presence of the transgene cDNAs in the infected tumors was not related to a higher transgene stability, but rather to nonreplicative proviral sequences containing intact transgene cDNAs which persisted in tumors through the proliferation of cells carrying defective proviruses. Transcripts arising from both oFV promoters, including the transgene and the genomic RNA transcripts, were detected in the infected tumors (Fig. 5C) suggesting that viral replication does occur in the tumor to a certain extent. However, further analysis is required to determine if the transgene-expressing proviruses in the infected tumors give rise to infectious particles. FV genome instability during in vitro amplification has been well documented and it results in deletions in the U3 region of the FV LTR [42, 43]. Interestingly, an analysis of SFV isolates from zoonotically humans revealed that these deletions are rarely found for genomes amplified in vivo [9]. In fact, SFV genomes have been shown to be highly stable during long term in vivo infections, showing less than 1% variation over 13 years [44]. It is possible, therefore, that during in vivo replication variants carrying deletions in transgene cDNAs do not necessarily have selective advantage over the parental vectors, thus the parental viruses are not rapidly outgrown by the deletion variants. Intriguingly, a PCR analysis of the in vivo stability of CD in Toca 511, which showed superior in vitro stability, revealed that while the intact transgene cDNA was abundant in the infected tumors, various deletion events were detected upon intratumoral amplification over the period of 38 days [16]. The oFV-TK vector showed a comparable in vivo stability upon intratumoral amplification for 46 days despite its significant in vitro instability.

The loss of the iCasp9 cDNA together with the apparently higher stability of the GFP cDNA during in vivo amplification of the vectors suggest that transgene size might impact its stability in the viral backbone. As discussed above, it is unlikely that the higher instability of certain transgenes is caused by a limited genome packaging capacity. However, it is likely that both the transgene size and the nature of the foreign sequences impact genome stability. The RNA genome of HIV-1 forms highly organized secondary structures [45], which were demonstrated to regulate splice site use and viral gene expression [46]. Such secondary structures have not yet been identified for the FV RNA genome, however, it is very likely that they exist and play important roles in the viral life cycle. Therefore, introduction of certain, especially long foreign sequences into the viral genome might destabilize the formation of the secondary structures, affecting processes in the viral life cycle that revolve around the RNA genome, including genome and Pol packaging, reverse transcription, splicing or translation. As a result, these burdensome sequences dispensable for viral replication are deleted from the viral genome, the deletion variants have a selective advantage over the viruses with intact transgene cDNA. The seemingly stable expression of GFP in the oFV-GFP infected tumors may indicate that smaller transgenes could have a lesser destabilizing effect on the viral replication and their loss does not confer a strong selective advantage but may equally reflect on the lack of toxic secondary RNA structure in the GFP transgene. Therefore, oFV vectors armed with proteins encoded by short cDNAs could be more stable than the TK and iCasp9 vectors. This hypothesis will be thoroughly addressed in future studies by evaluating the stability and function of well-characterized small transgene cDNAs, such antitumor response modulating cytokines GM-CSF, IL-2 and IL-15, encoded within the oFV backbone.

Deletions identified in the coding region of the FV genome, specifically within the *tas* gene of PFV therefore leading to defective proviral DNA, have been linked to occasional packaging of partially spliced RNA genomes during replication both in vitro and in vivo [47]. The deletion events leading to transgene loss in our oFV vectors could occur by a similar mechanism, therefore we investigated if transgene cDNA deletion is associated with a specific splicing event. Identification of splice donors and acceptors leading to this splicing event could allow for their inactivation and potential improvement of transgene stability. Our results showed that deletions occurred in a random manner (Supplementary Figs. 2, 3b, 4b), with none of the analyzed clones sharing the same breaking points in their genome sequences. Moreover, these breaking points did not match the potential cryptic splice donors and acceptors identified in the transgene sequences (data not shown), indicating that the transgene loss might not occur in a typical splicing event and the mechanism needs to be further investigated. Optimization of oFV vector design by elimination of cryptic splice sites and investigating alternative transgene insertion sites in the viral genome should nevertheless be explored as it might increase the stability of the oFV vectors.

The transient decrease in bioluminescence in the oFV-iCasp9 infected indicator tumors upon intraperitoneal AP20187 treatment (Figs. 3C, D) is likely a result of low expression of the transgene caused transgene loss, as following tumor explantation the indicator cells were insensitive to in vitro AP20187 treatment (Supplementary Fig. 4a) and intact iCasp9 cDNA was not detected in those cells (Fig. 4D). However, the incomplete killing of oFV-TK infected tumor cells upon GCV treatment (Figs. 3A, B) seems unlikely to be caused by transgene instability as, despite some deletion events, all of the GCV treated, oFV-TK infected tumors carried intact TK cDNA (Fig. 4B). One possible explanation for this in vivo persistence of the oFV vector after GCV treatment is proviral latency preventing the expression of TK, as FVs have been shown to latently infect various tissues [13, 48]. This hypothesis could be supported by the finding that explanted and in vitro expanded oFV-TK infected indicator tumor cells died upon in vitro GCV treatment (Supplementary Fig. 3a). Though the FV latency mechanism is poorly understood, it has been proposed that the process involves downregulation of Tas expression and upregulation of Bet expression, which would lead to silencing of the internal promoter and in consequence, due to the lack of the expression of the Tas transactivator protein, silencing of the LTR promoter [49]. However, oFV vectors do not express the Bet protein which is believed to downregulate the activity of the internal promoter and prevent Tas expression. In addition, Tas is highly expressed in the oFV-TK infected tumor cells persisting upon GCV treatment, as demonstrated by the imaging of Tas-induced bioluminescence in the infected tumors (Fig. 3B). It is possible, however, that intratumoral latency still occurs and follows a different mechanism, which has not been yet elucidated. Alternatively, the TK cDNA could be non-functional due to mutations arising in its sequence. Sequencing of the intact TK cDNA bands revealed the presence of a few G to A mutations (data not shown), a hallmark of the mutagenic activity of the APOBEC3 enzymes [50]. Given that G to A mutations caused by APOBEC3 proteins have been reported for SFV genomes isolated from zoonotically infected humans [51], and that oFV vectors lack the functional APOBEC3G-counteracting Bet protein, it is possible that this form of restriction led to introduction of premature stop codons in some TK cDNAs causing GCV insensitivity and viral persistence in a portion of oFV-TK infected xenograft tumors. The issue of persistence could possibly be moot in an immunocompetent animal model, as anti-viral and antitumor adaptive immune responses may be capable of eliminating oFV-infected tumor cells. In addition, TK/GCV suicide gene therapy was reported to be enhanced by T cell mediated immune responses and work more efficaciously in syngeneic tumor models compared to xenograft tumor models [52], potentially explaining why oFV-TK/GCV therapy was not superior to oFV-TK alone in prolonging the survival of mice bearing subcutaneous U251-U3-mCherry-U3-luc tumors (data not shown).

In summary, we have investigated the ability of oFV vectors to carry foreign transgenes and deliver them into tumor cells in vivo. Infection with oFV-GFP, -TK and -iCasp9 vectors led to robust transgene expression both in vitro and in vivo. The vectors efficiently propagated in indicator subcutaneous xenograft glioblastoma tumors, however, showed variable stability during the in vivo amplification. oFV-GFP infected tumors stably expressed GFP (the smallest of the tested transgenes) for at least 66 days without signs of substantial transgene loss. oFV-TK showed limited TK cDNA loss after 46 days of intratumoral propagation, whereas oFV-iCasp9 lost its transgene entirely. Though in this study we evaluated only three transgenes and evaluation of additional transgenes is underway, our results indicate that the stability of oFV vectors is variable and might be related to both the size of the carried transgene and the nature of its sequence, and that oFV vectors can deliver various foreign cargo into tumors in vivo and facilitate its long-term expression.

## Methods

### Cells

293 T (ATCC CRL-11268), U251 (kindly provided by Dr. Kah-Whye Peng, Mayo Clinic) and BHK-21 (ATCC CCL-10) cells were cultured in DMEM media supplemented with 10% Fetal Bovine Serum (FBS) and 1% streptomycin/penicillin. SKOV-3 (ATCC HTB-77) and HT-29 (ATCC HTB-38) cells were cultured in McCoy’s 5 A (Modified) Medium supplemented with 10% FBS and 1% streptomycin/penicillin. CT-26 (ATCC CRL-2638) cells were cultured in RPM-1640 medium supplemented with 10% FBS and 1% streptomycin/penicillin.

### Transgene insertion into the oFV backbone

The generation of the poFV-GFP plasmid has been described previously [33]. To generate poFV-TK we linearized the poFV-GFP backbone with BspEI and SacII restriction enzymes (the GFP cDNA was cut out), added a T2A sequence to the TK in a PCR reaction (using the following primers: Forward attacccgcggGAAGGACGGGGGAGCCTCCTGACATGTGGCGACGTGGAGGAGAATCCCGGACCCATGGCTTCGTACCCCTGCCA; Reverse: gccgcgaccggtTCAGTTAGCCTCCCCCATCT) and ligated with the linearized backbone upon PCR product digestion with BspEI and SacII restriction enzymes. iCasp9 cDNA was cloned into the poFV backbone by adding a T2A sequence as well as overhangs homologous to both ends of the linearized poFV-GFP plasmid in a PCR reaction (primers used for iCasp9: forward TTATAGAAATTACCCGCGGGAAGGACGGGGGAGCCTCCTGACATGTGGCGACGTGGAGGAGAATCCCGGACCCATGCTCGAGGGAGTGCAGGT, reverse: CTCTCTGAGGTCCGGATTAGTCGAGTGCGTAGTCTGG) and inserting it into the linearized poFV backbone using In-Fusion HD EcoDry Cloning Plus kit (Takara). The constructs were then sequenced to confirm the presence and correctness of the transgenes.

### Virus rescue

To rescue the oFV viruses, we transfected the infectious clones into 293 T cells using FuGene6 (Roche, Indianapolis, IN, USA) in a 10 cm dish. 2 and 4 days post-transfection the producer cells were split 1:2, transferred to 15 cm dishes and indicator BHK21-U3-mCherry cells were added to the culture. 6 days post transfection the media containing the viral supernatant was collected, the intracellular viral particles were then released from the cells by 2 cycles of freezing and thawing. Finally, the virus prep was filtered through a 0.45 µm syringe filter (Millipore) and concentrated by ultracentrifugation with the SW32Ti rotor (Beckman) and a 20% sucrose cushion. The pellet was re-suspended in PBS and stored at -80 °C.

### Lentiviral vectors

The second generation lentiviral vectors carrying mCherry driven by the PAN1 SFV U3 promoter and Puromycin resistance cassette driven by the ubiquitin promoter were described previously [33]. We also generated lentiviral vectors carrying Fluc DNA driven by the PAN1 SFV U3 promoter and neomycin resistance cassette driven by the PGK promoter. The vectors were produced by transient transfection of 293 T cells with the following plasmids: pHR-SIN (vector), p8.91 QV (Gag-Pol expression construct) and pMD-G (VSV-G expression construct) using FuGene6 (Roche) at a weight ratio of 2:1:1 of vector (Fig. 1D), Gag-Pol expression plasmid and VSV-G expression plasmid. The vectors were harvested 72 h after transfection, filtered through 0.45 µm filter membrane (Millipore) and either immediately used for transduction (of U251, BHK-21, HT-29 or CT-26 cells) or stored at -80 °C. Vector-transduced cells were selected with puromycin (3-7 μg/ml, for the U3-mCherry lentiviral vector) or neomycin (G418, 1.25-5 mg/ml, for the U3-luc lentiviral vectors)

### Virus titer determination

For titration of oFV vectors we used 10^5^ BHK-21-U3-mCherry cells per well in a 24-well plate and infected them with 0.2, 1, or 5 μl (or 5, 10 or 50 μl for not concentrated virus) of virus prep. 72 h postinfection the cells were harvested for a flow cytometry analysis to determine the percentage of mCherry positive cells.

Viral titers were calculated by multiplying the number of cells seeded for infection by the fraction of mCherry or GFP positive cells and volume of viral prep used for infection in ml.

### Flow cytometry

Cells were dispersed into single-cell suspension by incubation in Versene (Gibco) at 37 °C for 30 min, fixed in 4%PFA and analyzed using the LSR-II flow cytometer (BD Biosciences). Results were analyzed using the FlowJo software.

### Multistep growth curves

1 * 10^5^ BHK-21-U3-mCherry cells were seeded in a well of a 6 well plate. 3 h later, the cells were infected with indicated oFV vectors at MOI = 0.01. Every two days the cells were collected for a Flow Cytometry analysis of mCherry (or GFP) positive cells. Infected cells were passaged at a 1:3 ratio every 4 days. The analyses were done in duplicates in 2 independent experiments.

### In vitro luciferase assay

2 * 10^4^ U251-U3-mCherry-U3-Luciferase cells, U251-U3-mCherry-SFFV-Luciferase cells (constitutively express firefly luciferase from the Spleen Focus Forming Virus (SFFV) promoter) or wildtype U251 cells were seeded per well of a flat, transparent bottom, black 96-well plate. The cells were infected with oFV at MOIs 0, 0.2, 1 and 5. On day 3 postinfection 100 μl of luciferin at 0.2 mg/ml was added to each well. Bioluminescence was then measured with Tecan Infinite M200 Pro and the results are shown as Relative Light Units (RLU). The experiment was done twice in duplicates.

### Transgene stability analysis

10^5^ U251-U3-mCherry cells in a 6-well plate were infected with oFV-TK or oFV-iCasp9 at MOI = 0.5. 4 days postinfection the infected cells were passaged 1:3. 8 days postinfection the infected cells were collected for total DNA isolation while the supernatants were filtered through a 0.45 µm syringe filter (Millipore), titrated and used to infect 10^5^ U251-U3-mCherry cells at MOI = 0.5. This process was repeated 5 times (5 cell-free passages of the viruses). Genomic DNA was isolated from the oFV-infected cells at the end of each passage with the DNeasy Blood & Tissue Kits (Qiagen). To detect the presence of the oFV provirus in the DNA samples and verify the intactness of the transgene cDNAs a PCR reaction was performed using primes binding within env of oFV (forward: GGATGGACCTCCAAACAAAT, reverse: AACCCAATTTCCCAAGCCGT). To detect the transgene a PCR reaction was ran using primes binding to the remaining sequences of *bel2* upstream and downstream of the transgene insertion site (forward: TGTCAGGAGGACCCTTCTGG, reverse: CTGGAGTATTTGGGTAGTGA). The PCR products whose size was smaller than the anticipated intact transgene cDNA (the deletion variants) were cut out, purified, cloned using the Zero Blunt™ TOPO™ PCR Cloning Kit for Sequencing system (Invitrogen) and sequenced. Subsequently, the sequences were analyzed and aligned with the reference sequences using DNADynamo (BlueTractorSoftware Ltd).

### In vitro prodrug treatment

2 * 10^4^ target cancer cells were seeded per well of a 96 well plate and infected with oFV-TK or oFV-iCasp9, or oFV-GFP at MOI = 1 or left uninfected. Three to five days postinfection, depending on the cell line, the cells were treated with GCV (20 μM) or AP20187 (100 nM) or mock control. Efficacy of the oFV-TK/GCV combination was measured by viability measurement with PrestoBlue (Invitrogen) for up to 7 days post GCV treatment. Results are shown as percent untreated control. Efficacy of the oFV-iCasp9/AP20187 combination was measured with a flow cytometry analysis of live, mCherry positive (oFV-infected) indicator cancer cells 24 h post-AP20187 treatment.

### In vivo experiments

All animal experiments were approved by Mayo Clinic’s Institutional Animal Care and Use Committee. Six-week-old female CB-17 SCID mice obtained from the vendor Envigo were injected subcutaneously in the right flank with 5 * 10^6^ U251-U3-mCherry-Luciferase cells. No randomization or blinding was used. When tumors reached the volume of 0.3–0.5 cm^3^, they were directly injected with four doses of 5 * 10^5^ IU of oFV-GFP (*n* = 5), 5 doses of 2 * 10^6^ IU oFV-TK (*n* = 20) or 5 doses of 2 * 10^6^ IU oFV-iCasp9 (*n* = 20) in 100 μl PBS or 100 μl of PBS (control, *n* = 10 for oFV-TK study and *n* = 10 for oFV-iCasp9 study). The mice were weighed and tumor volumes measured three times a week. The mice were followed for up to 102 days after the first injection unless they reached end-point conditions based on the tumor size (greater than 10% body weight) or body scoring condition and were euthanized. Once a week, the mice were anesthetized and imaged with Xenogen IVIS-200 system after an intraperitoneal injection of luciferin (20 mg/ml).

oFV-TK infected mice (*n* = 10) were treated with Ganciclovir sodium (Selleck Chemicals LLC) at 50 mg/kg once a day, starting 14 days post first virus injection, for 4 five-day cycles. oFV-iCasp9 infected mice (*n* = 10) were treated with AP20187 (Takara) at 5 mg/kg once a day, starting 15 days postfirst virus infection, for 4 days. The sample size of 10 animals per group was chosen based on discussions with our statisticians anticipating a large effect size.

For tumor explantation, upon euthanasia tumors were minced, incubated with a solution containing type III collagenase (100 U/ml) and 3 mM CaCl2 for 2 h at 37 °C on a rocking platform. Then the samples were passed through a cell strainer, concentrated by low-speed centrifugation, re-suspended in 10% FBS DMEM media and plated in 6-well plates. To detect the presence of the oFV provirus in the tumor explants, genomic DNA was isolated from the explanted cultures with the DNeasy Blood & Tissue Kits (Qiagen). To detect the presence of the oFV provirus and transgenes in those tissues a PCR reaction was performed using primes binding within *env* of oFV and to the remaining sequences of *bel2* upstream and downstream of the transgene insertion site, as described above.

### Immunohistochemistry

Upon euthanasia, tumors were covered in OCT and flash frozen on dry ice. Tumors were then sectioned, fixed in 4% PFA, incubated overnight at 4 °C with primary antibodies at 1:100 dilution: anti-mCherry (chicken, polyclonal, Abcam) and anti-GFP (rabbit, polyclonal, Abcam). Then, the sections were stained with anti-chicken or anti-rabbit secondary antibodies conjugated with fluorophores, respectively, Alexa 594 and Alexa 488 (1:2000 dilution). The sections were imaged using Zeiss LSM 510 Confocal Microscope and analyzed with the Zen software.

### oFV-GFP gene expression analysis in infected tumors

oFV-GFP infected tumors were harvested 13 and 39 days postinfection together with a PBS-injected control and flash frozen on dry ice in OCT. Tumor fragments were then pulverized with TissueLyser II (Qiagen) and stainless beads (Qiagen), and total RNA was isolated from the tumors with RNeasy Plus Universal Mini Kit (Qiagen). The RNA was then sent for an RNAseq analysis to Admera Health (126 Corporate Blvd, South Plainfield, NJ 07080). The results were analyzed by the Mayo Clinic’s Division of Biomedical Statistics and Informatics using the genomic sequence of oFV-GFP and presented as Reads Per Kilobase of transcript, per Million mapped reads (RPKM).

### qPCR analysis

For the quantification of the GFP, TK and *env* copy numbers we performed qPCR on 50 ng of DNA isolated from oFV-GFP-infected, oFV-TK-infected or uninfected U251-U3-mChery-U3-luc tumors or in vitro infected U251-U3-mChery-U3-luc cells (total DNA isolation performed with DNeasy Blood & Tissue Kits, Qiagen) with TaqMan™ Universal PCR Master Mix (Applied Biosystems) and the ViiA™ 7 Real-Time PCR System. For GFP copy number quantification we used previously described primers (Forward: CCACATGAAGCAGCAGGACTT and Reverse GGTGCGCTCCTGGACGTA) and probe (56-FAM/TTCAAGTCC/ZEN/GCCATGCCCGAA/3IABkFQ) [53]. For the quantification of env we used the following primers: Forward: GTCACTCAGAGGGCTGTTTATC, Reverse: CTTGTGGGATACTGGTCATGT, Probe: /56-FAM/CGTTCCCTT/ZEN/AGAGTGCAACACCCA/3IABkFQ/. For TK copy number quantification we used the following primers: Forward: GTACCCGAGCCGATGACTTACT, Reverse: CCCGGCCGATATCTCA, Probe: /56-FAM/CTTCCGAGA/ZEN/CAATCGCGAACATCTACACC/3IABkFQ/. These primers have been described previously [54]. The analysis was performed relative to the β-Actin copy number using Prime Time Std qPCR assay Hs.PT.56a.40703009.g, (Integrated DNA Technologies).

### Statistical analysis

All statistical analyses were done using GraphPad Prism 8, with tests specified in figure legends and alpha=0.05. To our knowledge, the tests used in this study are appropriate and meet the necessary criteria. P values, sample sizes, number of replicates are reported in figures and figure legends.

## Supplementary information

Supplementary figure legends

Supplementary figure 1

Supplementary figure 2

Supplementary figure 3

Supplementary figure 4

Supplementary figure 5

## Data Availability

The data generated and analyzed during this study can be found within the published article and its supplementary files.
