# Supplementary material for: Evaluation of the stability and intratumoral delivery of foreign transgenes encoded by an oncolytic Foamy Virus vector
Source: Cancer Gene Ther. 2022 Feb 10;29(8-9):1240–51. doi: 10.1038/s41417-022-00431-y (PMC9363555; doi:10.1038/s41417-022-00431-y)
Supplement: Supplementary file 1 — Supplementary figure legends [file 41417_2022_431_MOESM1_ESM.docx]

## **Supplementary figure legends**

**Supplementary figure 1 *In vitro* infection of cancer cell lines with oFV vectors carrying TK and iCasp9 renders the infected cells susceptible to GCV/AP20187 treatment:** Fluorescence microscope imaging of indicator U251-U3-mCherry-U3-luc cells infected with an oFV control and oFV-TK (A) or oFV-iCasp9 (C) at MOI=1 and treated with an appropriate prodrug or a mock control, B. Viability of U251-U3-mCherry and SKOV-3 cancer cell lines mock infected or infected with oFV-GFP or oFV-TK at MOI=1 and treated with 20μM GCV or mock control (results presented as percent of mock-treated control, experiment performed in triplicates, statistical significance determined using the Holm-Sidak method), D. Percent of mCherry positive cells after mock, oFV-GFP or oFV-iCasp9 infection at MOI=1 and treatment with AP20187 or mock control in HT-29-U3-mCherry indicator cancer cell line (measured 24 hours post AP20187 treatment, statistical significance determined using the Holm-Sidak method, performed in triplicates).

**Supplementary figure 2 Deletion events leading to the loss of transgenes in the oFV backbone occur in a random manner:** Dropout bands from the experiment in Fig. 4a (indicating deletion events in the transgene) from were cut out, gel purified and TOPO-cloned (Zero Blunt™ TOPO™ PCR Cloning Kit for Sequencing), then sequenced and aligned (DNADynamo, BlueTractorSoftware Ltd) with the transgene-containing part of the oFV genome to determine the breaking points for TK (A) and iCasp9 (B); TK c.1 (clone 1) and c. 4 were derived from the dropout band at approximately 55bp, c.2 was derived from the bad at approximately 700 bp, c.3 was derived from the band at approximately 1kb. For iCasp9, (B), c.1 and c.3 were derived from the dropout band at approximately 700 bp, c2. Was derived from the band at 1.5 kb, c.4 was derived from the dropout band at approximately 750 bp. iCasp9 TK/iCasp9 start/stop in green font- beginning and end of the transgene ORF.

**Supplementary figure 3 GCV treatment of explanted and *in vitro* expanded oFV-TK-infected U251-U3-mCherry-U3-Luc tumors leads to cell death, transgene loss during *in vivo* amplification occurs in a random manner** A. U251-U3-mCherry-U3-luc tumors from mice infected with oFV-TK and treated with GCV or PBS control were explanted and cultured *in vitro*, the explants were treated with GCV or mock control and imaged with fluorescent microscope to verify if the explants are sensitive to GCV, B. The dropout bands from the experiment in Fig. 4b (indicating deletion events in the transgene) were cut out, gel purified and TOPO-cloned, sequenced and aligned with the TK containing part of the oFV genome to determine the breaking points in the transgene; c.1 and c.3 were derived from the dropout band at approximately 400 bp, c.2 was derived from the droput band at approximately 900 bp,TK start/stop - beginning and end of the transgene ORF.

**Supplementary figure 4 Explanted and *in vitro* expanded oFV-iCasp9-infected U251-U3-mCherry-U3-Luc tumor cells are insensitive to AP20187 treatment, transgene loss during *in vivo* amplification occurs in a random manner**. U251-U3-mCherry-U3-luc tumors from mice infected with oFV-iCasp9 and treated with AP20187 or PBS control were explanted and cultured *in vitro,* the explants were treated with AP20187 or mock control and imaged with fluorescent microscope to verify if the explants are sensitive to AP20187, B. The dropout bands from the experiment in Fig. 4d (indicating deletion events in the iCasp9 transgene) were cut out, gel purified and TOPO-cloned, sequenced and aligned with the iCasp9 containing part of the oFV genome to determine the breaking points in the transgene; c.1 was derived from the dropout band at 1kb, c.2 and c.3 were derived from the dropout band at 700bp, iCasp9 start/stop - beginning and end of the transgene ORF.

**Supplemental figure 5 oFV-GFP efficiently propagates in U251-U3-mCherry-U3-Luc tumors with minor signs of transgene loss:** 5 million U251-U3-mCherry-U3-luc cells were implanted subcutaneously in CB17-SCID mice and infected intratumorally with PBS control or 4 doses of 5*10*^5^* IU oFV-GFP, A. bioluminescence imaging of oFV-GFP infected tumors at 3 different time points, B. Immunofluorescence staining for mCherry and GFP in sections of oFV-GFP infected tumors, regions positive only for mCherry (indicating transgene loss) were rarely found in tumors at 66 days post infection.
